# *Arachis hypogaea resveratrol synthase 3* alters the expression pattern of *UDP-glycosyltransferase* genes in developing rice seeds

**DOI:** 10.1371/journal.pone.0245446

**Published:** 2021-01-14

**Authors:** Choonseok Lee, Woo-Jong Hong, Ki-Hong Jung, Ha-Cheol Hong, Dool-Yi Kim, Hyun-Choong Ok, Man-Soo Choi, Soo-Kwon Park, Jaehyun Kim, Hee-Jong Koh

**Affiliations:** 1 Department of Plant Science, Research Institute for Agriculture and Life Sciences, and Plant Genomics and Breeding Institute, Seoul National University, Seoul, Republic of Korea; 2 Graduate School of Biotechnology, Kyung Hee University, Yongin, Gyeonggi-do, Republic of Korea; 3 National Institute of Agricultural Sciences, Wanju, Jeollabuk-do, Republic of Korea; 4 National Institute of Crop Science, Wanju, Jeollabuk-do, Republic of Korea; 5 Rural Development Administration, Jeonju, Jeollabuk-do, Republic of Korea; Universidade de Lisboa Instituto Superior de Agronomia, PORTUGAL

## Abstract

The resveratrol-producing rice (*Oryza sativa* L.) inbred lines, Iksan 515 (I.515) and Iksan 526 (I.526), developed by the expression of the groundnut (*Arachis hypogaea*) *resveratrol synthase 3* (*AhRS3*) gene in the *japonica* rice cultivar Dongjin, accumulated both resveratrol and its glucoside, piceid, in seeds. Here, we investigated the effect of the *AhRS3* transgene on the expression of endogenous piceid biosynthesis genes (*UGTs*) in the developing seeds of the resveratrol-producing rice inbred lines. Ultra-performance liquid chromatography (UPLC) analysis revealed that I.526 accumulates significantly higher resveratrol and piceid in seeds than those in I.515 seeds and, in I.526 seeds, the biosynthesis of resveratrol and piceid reached peak levels at 41 days after heading (DAH) and 20 DAH, respectively. Furthermore, RNA-seq analysis showed that the expression patterns of *UGT* genes differed significantly between the 20 DAH seeds of I.526 and those of Dongjin. Quantitative real-time PCR (RT-qPCR) analyses confirmed the data from RNA-seq analysis in seeds of Dongjin, I.515 and I.526, respectively, at 9 DAH, and in seeds of Dongjin and I.526, respectively, at 20 DAH. A total of 245 *UGT*s, classified into 31 UGT families, showed differential expression between Dongjin and I.526 seeds at 20 DAH. Of these, 43 *UGT*s showed more than 2-fold higher expression in I.526 seeds than in Dongjin seeds. In addition, the expression of resveratrol biosynthesis genes (*PAL*, *C4H* and *4CL*) was also differentially expressed between Dongjin and I.526 developing seeds. Collectively, these data suggest that *AhRS3* altered the expression pattern of *UGT* genes, and *PAL*, *C4H* and *4CL* in developing rice seeds.

## Introduction

Resveratrol (3,5,4'-trihydroxystilbene), a stilbene compound, occurs in many plant species including grape (*Vitis vinifera*), groundnut (*Arachis hypogaea*), *Eucalyptus* spp., Texas fescue (*Festuca versuta*) and Japanese knotweed (*Polygonum cuspidatum*) [1–8]. The glucoside of resveratrol, piceid (3,5,4'-trihydroxystilbene-3-*β*-monoglucoside), has also been detected in several plant species, either with or without resveratrol, such as *Eucalyptus* spp., grape, *Picea* spp. and *P*. *cuspidatum* [3,5,6,9–13]. Both resveratrol and piceid play various physiological roles in animals [14–20]. Resveratrol exhibits antineoplastic, cardioprotective and antioxidant properties in activated blood platelets [14–16] and inhibits low-density lipoprotein (LDL) peroxidation [17]. Studies in mice and rats show that piceid is involved in the improvement of renal ischemia/reperfusion injury, neuroprotection, and cardiomyocyte protection [18–20]. *In planta* metabolic engineering of the *resveratrol synthase* (*RS*), a gene encoding stilbene synthase that catalyzes the biosynthesis of resveratrol from one molecule of *p*-coumaroyl-CoA and three molecules of malonyl-CoA [21–25], has been conducted in many plant species to develop cultivars with high resveratrol levels for human health benefits [26–30].

Iksan 515 (I.515) and Iksan 526 (I.526) are two resveratrol-producing inbred lines of rice (*Oryza sativa* L. subsp. *japonica* cv. Dongjin) carrying the groundnut *resveratrol synthase 3* (*AhRS3*; DQ124938) gene [29,31,32]. These inbred lines were generated by Dr. Baek’s research team at the National Institute of Crop Science (NICS), Republic of Korea, using the *Agrobacterium*-mediated transformation method [29,31,32]. Both resveratrol and piceid were detected in the leaves and seeds of I.526 [29,32], although the quantity of piceid in leaves was significantly higher than that in seeds. Like I.526, tomato (*Lycopersicon esculentum*) fruits of transgenic lines overexpressing the grape *stilbene synthase* gene produced both resveratrol and piceid [26,28]. Interestingly, only piceid was detected in the transgenic lines of other plant species, such as alfalfa (*Medicago sativa*), apple (*Malus domestica*), poplar (*Populus alba*), *Brassica napus* and *Arabidopsis thaliana*, overexpressing the *stilbene synthase* gene of grape or the *RS* gene of groundnut or *P*. *cuspidatum* [33–37].

**Fig 1:**
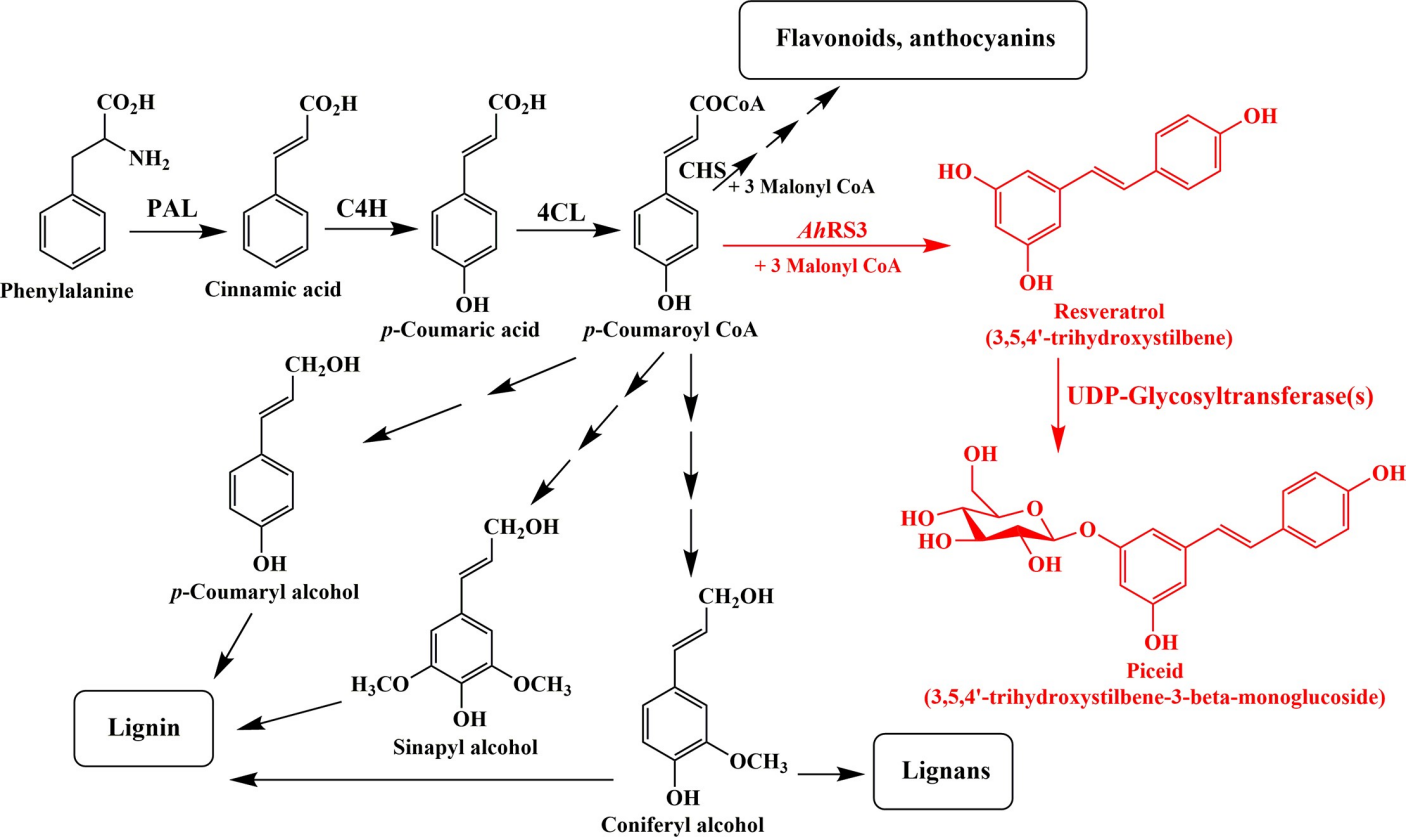
Scheme of biosynthetic pathway of resveratrol and piceid. PAL, phenylalanine ammonia-lyase; C4H, cinnamate 4-hydroxylase; 4CL, 4-coumarate: CoA ligase; CHS, chalcone synthase; *Ah*RS3, *Arachis hypogaea* resveratrol synthase 3. The pathway shown in red is based on the results obtained from the developing seeds of I.526.

A schematic biosynthetic pathway of the resveratrol and piceid in the developing seeds of I.526 is shown in Fig 1. In this pathway, phenylalanine is converted to *p*-coumaroyl CoA in a three-step process by the action of phenylalanine ammonia-lyase (PAL) [38], cinnamate 4-hydroxylase (C4H) [39] and 4-coumarate: CoA ligase (4CL) [40]. Then, *p*-coumaroyl CoA is converted to resveratrol by *Ah*RS3 upon the addition of malonyl-CoA [24,29]. Piceid is readily generated from resveratrol by the action of UDP-glycosyltransferase(s) (UGTs). This is consistent with previous reports on the metabolic engineering of resveratrol in other plant species [26,28,33–37]; however, the role of *UGT* genes in resveratrol biosynthesis has not been reported previously, except for the bi-functional *resveratrol/hydroxycinnamic acid glucosyltransferase* gene reported in Concord grape (*Vitis labrusca*) [41] (Fig 1).

In this study, we investigated the number of *UGT* genes whose expression levels were affected by the introduction of *AhRS3*, with the aim to identify candidate *UGT* gene(s) involved in piceid biosynthesis. In addition, we examined whether the expression patterns of genes acting upstream of *AhRS3*, including *PAL*, *C4H* and *4CL*, were altered by the introduction of *AhRS3* in the developing seeds of I.526.

## Materials and methods

### Growth of resveratrol-producing transgenic rice lines

To develop resveratrol-producing transgenic rice lines, *AhRS3* (DQ124938) was cloned from groundnut (*A*. *hypogaea*) and inserted into pSB2220 vector carrying the maize *Ubi1* promoter for overexpression of a target gene [29]. *Agrobacterium tumefaciens* strain LBA4404 carrying pSB2220 vector with *AhRS3*, was cocultivated with calli derived from seeds of Dongjin, a japonica rice cultivar (*O*. *sativa* L. subsp. *japonica* cv. Dongjin), to generate transgenic calli. Transgenic rice plants were regenerated from transgenic calli selected. These works were performed by Dr. Baek’s research team at the NICS, Republic of Korea [29].

Seedlings of wild-type rice (*Oryza sativa* subsp. *japonica* cv. Dongjin) and inbred lines of its transgenic counterpart, I.515 and I.526, were transplanted in the genetically modified organism (GMO) greenhouse (30 cm × 15 cm) of the National Institute of Crop Science (NICS), Republic of Korea, using the standard rice cultivation method of the NICS. A completely randomized design (CRD) was used, with three replications. Developing seeds were harvested from one panicle per plant (total four plants per replication) at 9 days after heading (DAH).

The seedlings of wild-type rice and I.526 were transplanted in the GMO paddy field (30 cm × 15 cm) of the NICS, Republic of Korea, using the standard rice cultivation method of the NICS. As mentioned above, a CRD was used, with three replications. Developing seeds were harvested from one panicle per plant (total four plants per replication) at 6, 13, 20, 31 and 41 days after heading (DAH).

### Quantification of resveratrol and piceid by UPLC

Seed extracts were prepared from each sample, as described previously [29], and 1-μl aliquots of each sample were analyzed using the ACQUITY UPLC system (Waters Corporation, Milford, MA, USA) with ACQUITY UPLC BEH C18 column (1.7 μm, 2.1 × 100 mm; Waters Corporation, Milford, MA, USA). Resveratrol was analyzed over a total run time of 30 min using acetonitrile (solvent A) and water (solvent B) as the mobile phase. The column was initially equilibrated with 10% A and 90% B. The ratio of A:B was gradually changed to 25:75 by 20 min from the initial time with curve7 and then to 100:0 (curve6) by 21 min. The A:B ratio was kept at 100:0 by 25 min with curve6, changed to 10:90 (curve6) by 26 min and then kept at 10:90 by 30 min with curve6. To analyze piceid, the column was initially equilibrated with 10% A and 90% B. The A:B ratio was gradually changed to 50:50 with curve9 by 20 min and then transformed to 100:0 with curve6 by 21 min. The UPLC analytical method for piceid from 21–30 min was same as that used for resveratrol, as described above. Both resveratrol and piceid were detected at 308 nm using the ACQUITY UPLC Tunable UV detector (Waters Corporation, Milford, MA, USA), and the flow rate was maintained at 0.2 ml/min.

Resveratrol and piceid standards were purchased from Sigma-Aldrich (Saint Louis, MO, USA). The UPLC solvents, water and acetonitrile were purchased from Thermo Fisher Scientific (Waltham, MA, USA).

### Total RNA extraction and RNA-seq

Total RNA was extracted from frozen and milled samples of developing seeds, including 9 DAH seeds of Dongjin, I.515 and I.526, and 6, 13, 20, 31 and 41 DAH seeds of Dongjin and I.526, using the RNeasy Plant Mini Kit (QIAGEN, Hilden, Germany), according to the manufacturer’s instructions. The total RNA samples of Dongjin and I.526 collected at 20 DAH seeds were sent to Macrogen, Inc. (Seoul, Republic of Korea) for RNA-seq using the Illumina technology.

Raw sequence reads were cleaned by removing low-quality nucleotides (Phred score < 20) and short sequence reads (read length < 20 nt). The cleaned reads were mapped onto the Nipponbare reference genome sequence retrieved from the Rice Genome Annotation Project database (http://rice.plantbiology.msu.edu, version 7.0) using the HISAT2 software with default parameters [42]. The featureCounts software [43] was used to quantify the raw read counts from the BAM file, and normalized read counts were calculated by dividing the read counts of all genes with those of the *OsUBI1* gene. Raw data for RNA-seq are available at https://www.ebi.ac.uk/arrayexpress/experiments/E-MTAB-9695.

### Gene expression analysis by RT-qPCR

The isolated total RNA was quantified by NanoVue Plus (GE Healthcare Life Sciences, Chicago, IL, USA), and cDNA was synthesized from 1 μg of total RNA using the iScript^TM^ cDNA Synthesis Kit (Bio-Rad, Hercules, CA, USA). The primer sets used in the quantitative real time-polymerase chain reaction (RT-qPCR) are shown in S1 and S2 Tables. The rice *ACTIN1* gene (*OsACT1*; *LOC_Os03g50885*) was chosen as a reference (S1 Table). The RT-qPCR was carried out on the CFX96^TM^ Real-Time Detection System (Bio-Rad, Hercules, CA, USA) using iQ SYBR Green Supermix (Bio-Rad, Hercules, CA, USA). The relative expression of *AhRS3*, *UGT* genes, and genes acting upstream of *AhRS3*, including *PAL*, *C4H* and *4CL*, was determined using the Pfaffl method [44].

### Statistical analysis

All statistical analyses were performed using SAS 9.4 TS Level 1M5 (64-bit; SAS Institute Inc., Cary, NC, USA).

## Results

### Resveratrol and piceid biosynthesis in the developing seeds of resveratrol-producing rice inbred lines, Iksan 515 and Iksan526

**Fig 2:**
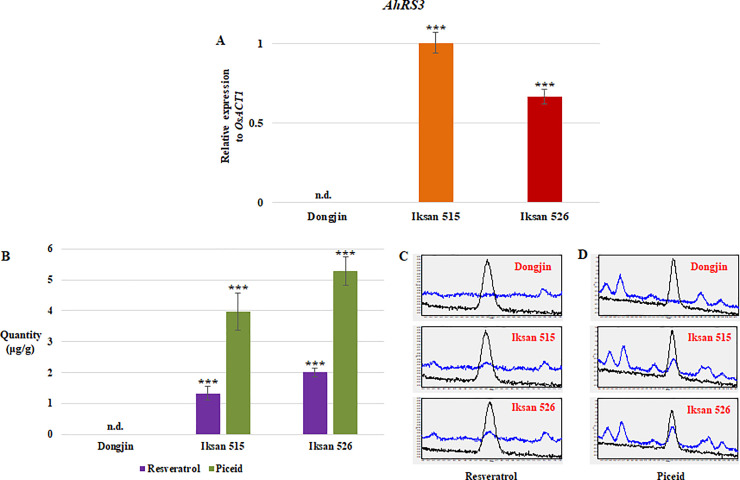
Analysis of *AhRS3* expression and resveratrol and piceid biosynthesis in the developing seeds of I.515 and I.526, respectively, at 9 days after heading (DAH). (A) Expression of *AhRS3* relative to that of the rice *ACTIN 1* (*OsACT1*) gene (internal control). (B) Levels of resveratrol piceid in 9 DAH seeds of I.515 and I.526, respectively. (C, D) Chromatogram from UPLC analysis of resveratrol (C) and piceid (D). Black line indicates the standard, and blue line indicates the extract of Dongjin, I.515 and I.526 seeds at 9 DAH. Data represent mean ± standard deviation (SD). ***: *p* < 0.001. n.d.: not detected.

The expression of *AhRS3* was confirmed in seeds of both I.515 and I.526 at 9 DAH, and the expression level of *AhRS3* was higher in 9 DAH seeds of I.515 than that in I.516 (Fig 2A). However, significantly higher quantities of resveratrol and piceid, respectively, were detected in 9 DAH seeds of I.526, compared to those of I.515 (Fig 2B–2D). Based on biosynthesis of resveratrol and piceid in developing seeds, I.526 was chosen for paddy field experiments to investigate biosynthesis of resveratrol and piceid, and gene expression analysis.

**Fig 3:**
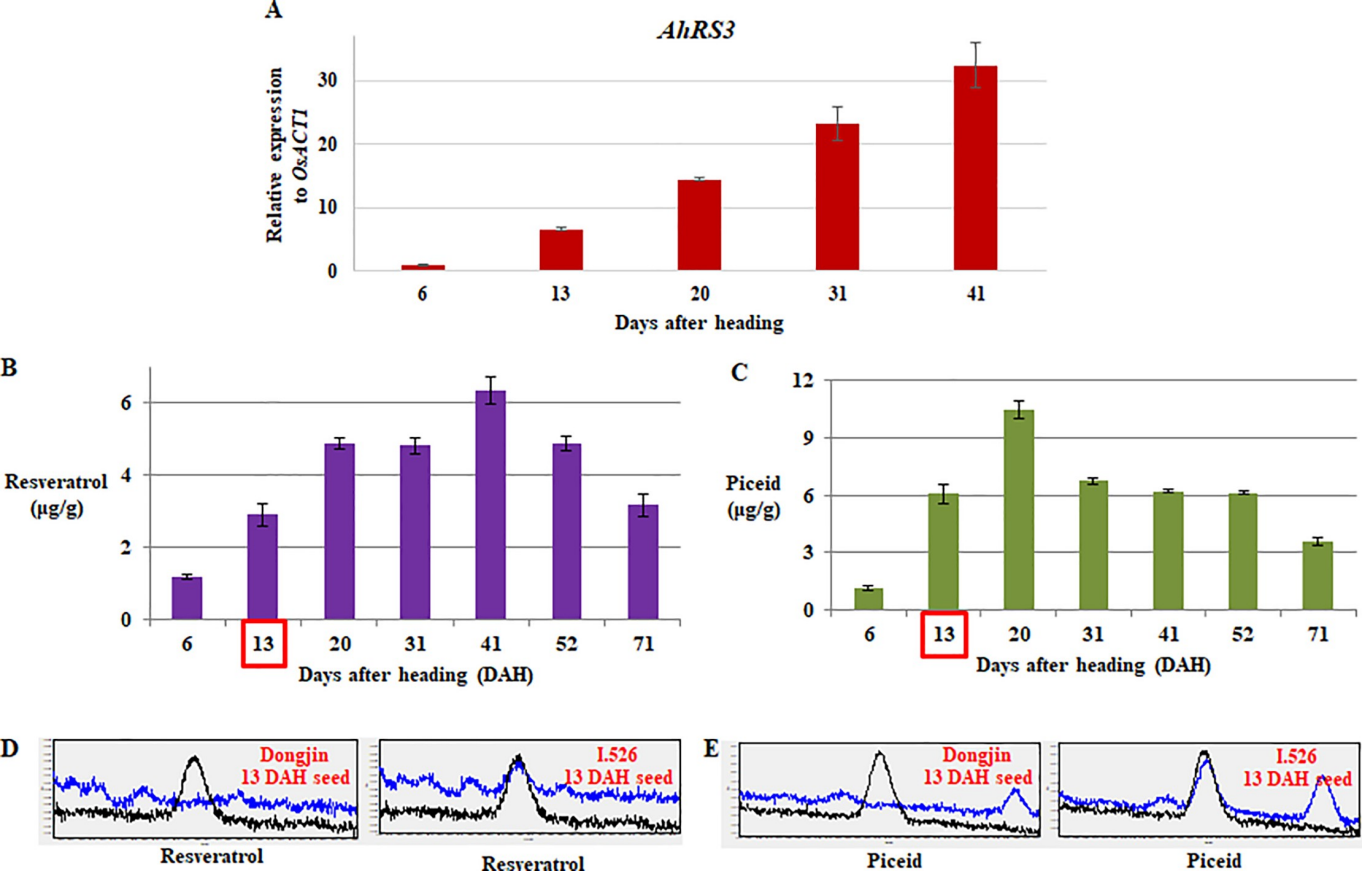
Analysis of *AhRS3* expression and resveratrol and piceid biosynthesis in the developing seeds of I.526 at different time points. (A) Expression of *AhRS3* relative to that of the rice *ACTIN 1* (*OsACT1*) gene (internal control). (B, C) Levels of resveratrol (B) piceid (C) in I.526 seeds. (D, E) Chromatogram from UPLC analysis of resveratrol (D) and piceid (E). Black line indicates the standard, and blue line indicates the extract of Dongjin and I.526 seeds at 13 days after heading (DAH). Data represent mean ± standard deviation (SD).

The expression level of *AhRS3* in I.526 seeds increased with development, reaching a peak at 41 days after heading (DAH) (Fig 3A). Ultra-performance liquid chromatography (UPLC; Waters Corp., Milford, MA, USA) analysis revealed that the biosynthesis of resveratrol and piceid in I.526 seeds increased initially and then decreased with development (Fig 3B–3E); however, both resveratrol and piceid reached maximal biosynthesis at different time points (41 and 20 DAH, respectively) (Fig 3B and 3C).

In addition, the expression of genes acting upstream of *AhRS3* in the resveratrol biosynthesis pathway was investigated by quantitative real-time PCR (RT-qPCR) analysis (S1 Fig). The relative expression levels of *PAL*, *C4H* and *4CL* were altered in developing seeds of I.526 compared with those in Dongjin. In I.526 seeds, all genes tested in this study, including *PAL*, *C4H* and *4CL*, showed typical expression patterns with significantly higher expression at 20, 31 and 41 DAH than those in Dongjin (S1 Fig).

### RNA-seq analysis of Dongjin and I.526 seeds

To analyze the transcriptome of the developing seeds of Dongjin and I.526, we performed RNA-seq analysis. Because UPLC analysis revealed that the glycosylation of resveratrol in I.526 seeds was the highest at 20 DAH (Fig 3C), we chose this time point for RNA-seq analysis. Raw sequence data of Dongjin and I.526 were mapped to the Nipponbare reference genome, and read counts of each gene were normalized relative to those of the rice *UBIQUITIN 1* (*OsUBI1*; *LOC_Os03g13170*) gene. A total of 245 *UGT* genes were selected from the normalized data (S2 Fig and S3 Table). Functional annotation of these genes using the Rice Genome Annotation Project (http://rice.plantbiology.msu.edu) and the National Center for Biotechnology Information (NCBI; https://www.ncbi.nlm.nih.gov/) databases revealed their putative roles in the glycosylation of several secondary metabolites, including cytokinin, anthocyanidin, indole-3-acetate, flavonoids, hydroquinone, *cis*-zeatin, limonoid, betanidin, *cyclo*-DOPA and *N*-hydroxythioamide. In I.526 seeds at 20 DAH, 43 out of 245 *UGT* genes were upregulated by more than 2-fold compared with Dongjin seeds, whereas 95 *UGT* genes showed extremely low expression (S2 Fig and S3 Table).

**Fig 4:**
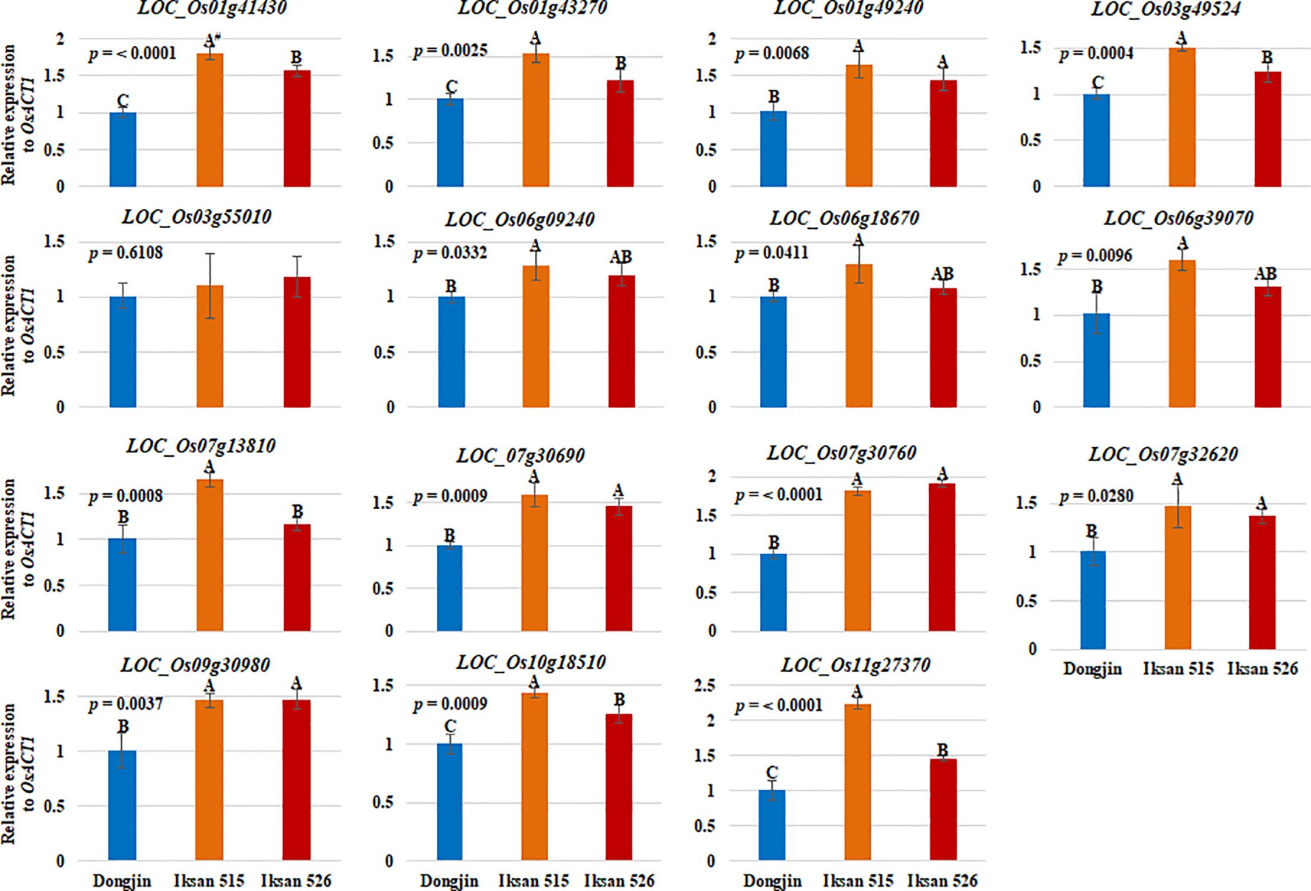
Verification of the expression level of selected *UGT*s (fold change ≥ 2), differentially expressed between Dongjin and I.526 seeds at 20 DAH, in 9 DAH seeds of Dongjin, I.515 and I.526, respectively, by RT-qPCR. Data represent mean ± standard deviation (SD). The *p*-values were obtained from the analysis of variance (ANOVA). ^#^: Duncan’s Multiple Range Test (DMRT), α = 0.05.

**Fig 5:**
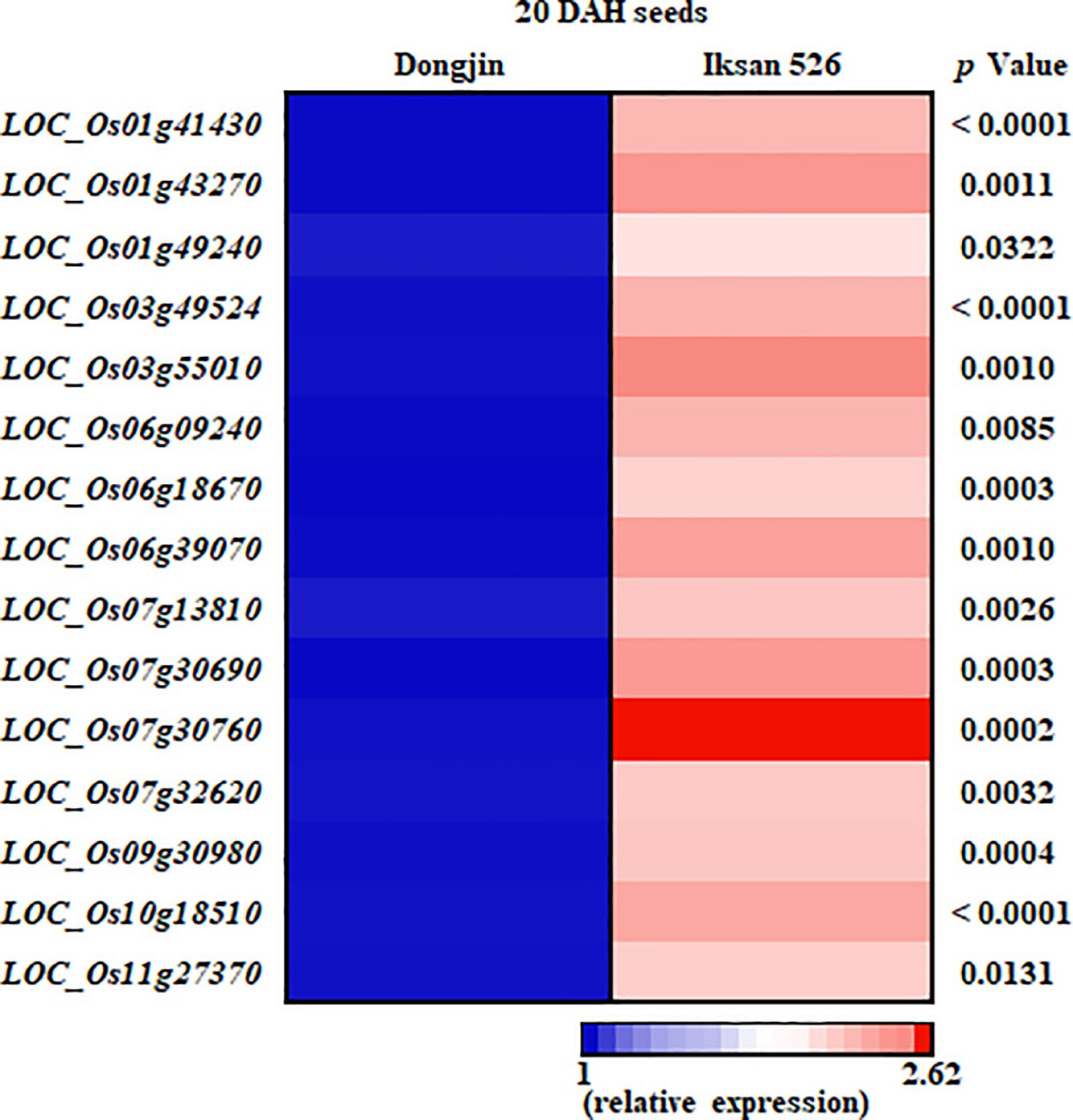
Verification of the expression level of selected *UGT*s (fold change ≥ 2) differentially expressed between Dongjin and I.526 seeds at 20 DAH by RT-qPCR. The *p*-values were obtained from the analysis of variance (ANOVA).

To verify the RNA-seq data, 15 out of 43 *UGT* genes upregulated in I.526 seeds were selected for RT-qPCR analysis (S2 and S3 Tables). The expression of all 15 *UGT* genes were first investigated by RT-qPCR analysis in seeds of Dongjin, I.515 and I.526 at 9 DAH, thereby resulting in consistency with the RNA-seq data, except for *LOC_Os03g55010* (Fig 4). In seeds of Dongjin and I.526 at 20 DAH, the results of RT-qPCR analysis of all 15 *UGT* genes were consistent with the RNA-seq data (Fig 5). Taken together, these results indicate that our RNA-seq data were highly reliable (Figs 4 and 5).

### Classification of the differentially expressed UGT genes

**Fig 6:**
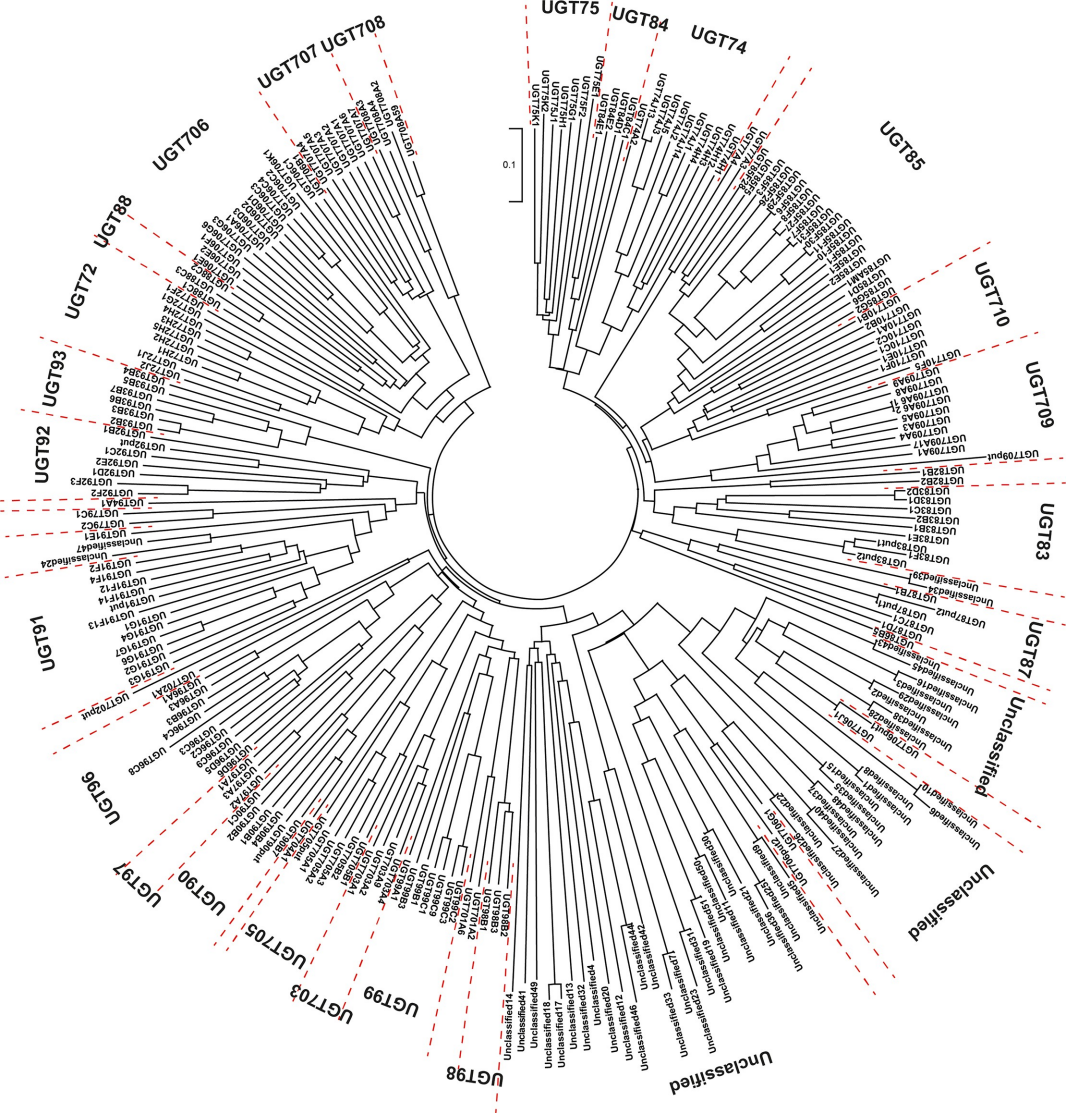
Phylogenetic analysis of 245 *UGTs* differentially expressed between Dongjin and I.526 seeds at 20 DAH.

Of the 245 differentially expressed *UGTs*, 186 genes encoded proteins containing more than 300 amino acids. The nomenclature of these 186 *UGTs* was determined by the UDP-glycosyltransferase (UGT) Nomenclature Committee (https://prime.vetmed.wsu.edu/resources/udp-glucuronsyltransferase-homepage) (S3 Table). Based on the nomenclature of these 186 *UGTs*, the public data obtained from the UGT Nomenclature Committee website and data retrieved from the NCBI, phylogenetic analysis of 245 UGTs was carried out using ClustalX 2.1 [45] and MEGA X [46] (Fig 6 and S3 Table). Of the 245 UGTs, 195 were classified into 31 families, while 50 remained unclassified. UGTs encoded by 43 *UGTs*, which showed >2-fold higher expression in I.526 seeds than in Dongjin seeds, were uniformly distributed among approximately 60% of the UGT families, including UGT72, UGT74, UGT75, UGT77, UGT79, UGT83, UGT84, UGT85, UGT90, UGT91, UGT93, UGT96, UGT97, UGT99, UGT703, UGT706, UGT707, UGT708, UGT709 and UGT710 (Fig 6 and S3 Table).

## Discussion

The *AhRS3* [21,24] gene, a missing link in the resveratrol biosynthesis pathway in rice, was successfully transformed into rice, resulting in the production of resveratrol [29] via the phenylpropanoid pathway, which produces *p*-coumaroyl CoA, a precursor of resveratrol [47] (Fig 1). In this study, RT-qPCR and UPLC analyses showed that *AhRS3* expression and resveratrol and piceid biosynthesis, respectively, increased in I.526 seeds with maturation, indicating that the biosynthesis of resveratrol is closely related to seed development (Fig 3). However, the expression of *AhRS3* and quantity of piceid in I.526 seeds were the highest at 41 and 20 DAH, respectively (Fig 3). This molecular phenotype might be related to expression patterns of *UGT*s (data not known) and availability of UDP-glucose in developing rice seeds [48]. Moreover, in seeds of black rice cultivars, the maximal biosynthesis of anthocyanins including cyanidin 3-glucoside and peonidin 3-glucoside was detected at 20 or 26 DAH (Lee et al., unpublished), consistent with the biosynthesis of piceid in developing seeds of I.526 (Fig 3).

In previous studies conducted on the metabolic engineering of resveratrol in tomato, piceid was detected along with resveratrol [26,28]; however, in genetically engineered plants of alfalfa, Arabidopsis, apple, poplar and *B*. *napus*, only piceid was detected [33]. This difference is probably related to endogenous UGTs, which are involved in the modification of secondary metabolites (to improve their stability and water solubility), inactivation and detoxification of xenobiotics, and regulation of hormones, including auxin, abscisic acid, cytokinins, brassinosteroids and salicylic acid [49].

Genes acting upstream of *AhRS3*, including *PAL*, *C4H* and *4CL*, were upregulated in the developing seeds of I.526 (S1 Fig). The upregulation of genes acting upstream or downstream of the target gene has been previously reported in the metabolic engineering of secondary metabolites *in planta* [50–54]. In addition, 245 *UGT*s showed differences in expression levels between I.526 and Dongjin seeds at 20 DAH (Fig 6 and S3 Table). All of these 245 UGTs were grouped into 31 UGT families. Of these, 43 *UGT* genes upregulated in I.256 seeds compared with Dongjin seeds (FC > 2) were classified into 20 UGT families, including UGT72, UGT74, UGT75, UGT77, UGT79, UGT83, UGT84, UGT85, UGT90, UGT91, UGT93, UGT96, UGT97, UGT99, UGT703, UGT706, UGT707, UGT708, UGT709 and UGT710 (Fig 6 and S3 Table). The biological roles of several UGTs have been revealed *in planta* (reviewed in Paquette et al. [55], Bowles et al. [56] and Bock [57]). For example, UGTs belonging to UGT71 [58], UGT74 [58], UGT76 [59], UGT78 [60], UGT79 [61], UGT84 [62], UGT88 [63], UGT90 [64], UGT95 [64], UGT707 [65] and UGT708 [66] families are involved in the biosynthesis of flavonoids. However, of all the *UGTs* reported to date, only one *UGT* gene (DQ832169) belonging to *V*. *labrusca* has been identified and characterized; this gene, which encodes resveratrol 3-*O*-glucosyltransferase, has been classified under the UGT84 family based on the UDP-glycosyltransferase Nomenclature Committee Website https://prime.vetmed.wsu.edu/resources/udp-glucuronsyltransferase-homepage) [41]. Furthermore, four differentially expressed *UGT* genes (*LOC_01g49240*, *LOC_Os01g49230*, *LOC_Os02g09510* and *LOC_Os05g47950*) were classified under the UGT84 family, one of which (*LOC_Os01g49240*) showed 2-fold higher expression in I.526 seeds than in Dongjin seeds (Fig 6 and S3 Table). However, the biological functions of these *UGTs* remain unknown. Therefore, biochemical and genetic analyses are needed to characterize the biological roles of these *UGTs in planta* and to determine whether other *UGT*s upregulated in I.526 seeds are involved in the glycosylation of resveratrol.

Overall, we showed that in the developing seeds of I.526, the time points of maximal biosynthesis of resveratrol and piceid were distinct, and *AhRS3* altered the expression pattern of *PAL*, *C4H*, *4CL*, and *UGT*s, resulting in the biosynthesis of resveratrol and piceid. Based on the expression data of target genes obtained by RNA-seq and RT-qPCR, we propose a model showing the positive regulation of upstream and downstream genes by *AhRS3* (S3 Fig).

## Supporting information

S1 Fig Relative expression of *PAL*, *C4H* and *4CL* in developing seeds of Dongjin and I.526 analyzed by RT-qPCR. The expression of all genes was normalized relative to that of *OsACT1*. Data represent mean ± standard deviation (SD). Asterisks indicate significant differences (*: 0.01 < *p* < 0.05; **: 0.001 < *p* < 0.01; ***: *p* < 0.001). ■: Dongjin, ■: I.526. N.A.: not applicable.
(TIF)

S2 Fig Identification of *UGT* genes differentially expressed between Dongjin and I.526 seeds by RNA-seq. (TIF)

S3 Fig Model showing *Ah*RS3-mediated positive regulation of upstream genes (*PAL*, *C4H* and *4CL*) and downstream genes (*UGT*s). (TIF)

S1 Table Primer sets for quantitative real time PCR (RT-qPCR). (DOCX)

S2 Table List of primers of *UGTs* used for RT-qPCR. (DOCX)

S3 Table List of 245 *UGTs* differentially expressed between Dongjin and I.526 seeds at 20 DAH. (XLSX)
