# Supplementary figures and images for: Arachis hypogaea resveratrol synthase 3 alters the expression pattern of UDP-glycosyltransferase genes in developing rice seeds
Source: PLoS One. 2021 Jan 14;16(1):e0245446. doi: 10.1371/journal.pone.0245446 (PMC7808588; doi:10.1371/journal.pone.0245446)

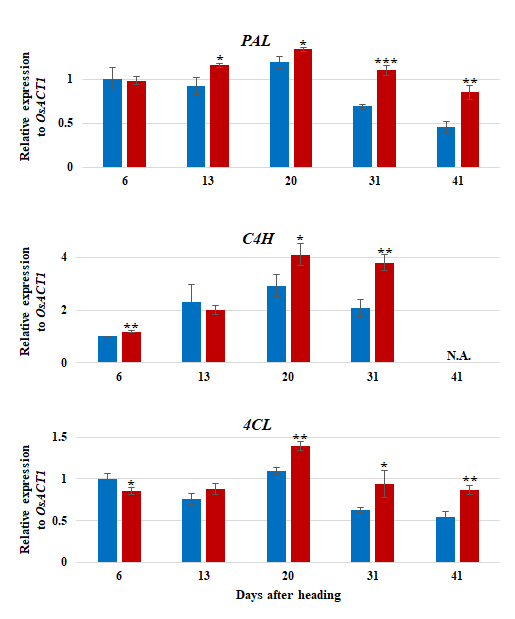

Supplement: S1 Fig — The expression of all genes was normalized relative to that of OsACT1. Data represent mean ± standard deviation (SD). Asterisks indicate significant differences (*: 0.01 < p < 0.05; **: 0.001 < p < 0.01; ***: p < 0.001). ■: Dongjin, ■: I.526. N.A.: not applicable. (TIF) [file pone.0245446.s001.tif]

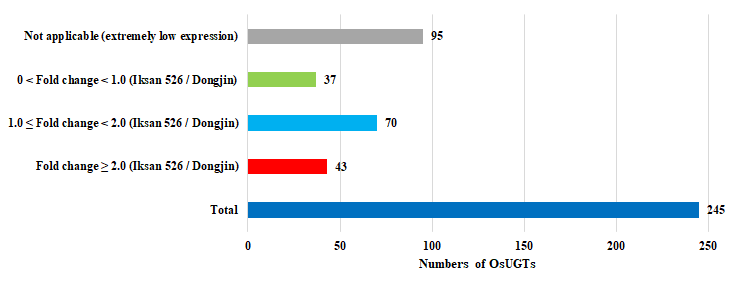

Supplement: S2 Fig — (TIF) [file pone.0245446.s002.tif]
