# Supplementary material for: Arachis hypogaea resveratrol synthase 3 alters the expression pattern of UDP-glycosyltransferase genes in developing rice seeds
Source: PLoS One. 2021 Jan 14;16(1):e0245446. doi: 10.1371/journal.pone.0245446 (PMC7808588; doi:10.1371/journal.pone.0245446)
Supplement: S1 Table — (DOCX) [file pone.0245446.s004.docx]

**S1 Table.** **Primer sets for quantitative real time PCR (qRT-PCR).**

| Gene name (Gene ID) | PCR Product size  (bp) | Forward (5' → 3') | Reverse (5' → 3') | Annealing T. (°C) |
| --- | --- | --- | --- | --- |
| *AhRS3* (DQ124938) | 160 | GGTAACATGTCAAGTGCATGTGTG | CCACACTGCGGAGCACAA | 56 |
| *PAL* (*LOC_Os02g41630*) | 160 | GTTCCCGCTCTACCGCTTC | GCCGTTCCACTCCTTGAGG | 57 |
| *C4H* (*LOC_Os05g25640*) | 160 | TCGCGCTGCCCATCAT | GCGTCGATGGGCTTGC | 58.5 |
| *4CL* (*LOC_Os02g08100*) | 160 | CGACCAAGAACACCATCGAC | GTGATGAGCAGCGCCTCC | 58 |
| *ACT* (*LOC_Os03g50885*) | 160 | ATCACTGCCTTGGCTCCTAGC | CAATGGATGGGCCAGACTCG | 58 |

*AhRS3*, *Arachis hypogaea Resveratrol synthase 3;* *PAL, Phenylalanine ammonia-lyase*; *C4H*, *Cinnamate 4-hydroxylase*; *4CL*, *4-Coumarate: CoA ligase*; *ACT*, *ACTIN* (*OsACT1*).
