# Supplementary material for: Arachis hypogaea resveratrol synthase 3 alters the expression pattern of UDP-glycosyltransferase genes in developing rice seeds
Source: PLoS One. 2021 Jan 14;16(1):e0245446. doi: 10.1371/journal.pone.0245446 (PMC7808588; doi:10.1371/journal.pone.0245446)
Supplement: S2 Table — (DOCX) [file pone.0245446.s005.docx]

**S2 Table. List of primers of *UGTs* used for qRT-PCR.**

| locus ID | Amplicon size (bp) | Forward (5'→3') | Reverse (5'→3') | Annealing  temperature  (°C) |
| --- | --- | --- | --- | --- |
| *LOC_Os01g41430* | 160 | GTGGAGGTTCTCAAGGTCGGG | CCGCTGCCTTCTTCCGTATC | 62 |
| *LOC_Os01g43270* | 160 | CTACTTCGGGACGAACGACCG | GTGCGACAAGAACCCTCCCA | 59 |
| *LOC_Os01g49240* | 160 | ACTTCCGACCAGGAAATGTGG | CGAGTGGACCGACCGGTATAA | 62 |
| *LOC_Os03g49524* | 160 | AACGGCGATGCGGAAAGCTA | GCCCGGACGCCTGATGAT | 59 |
| *LOC_Os03g55010* | 160 | TCTTCGACGCGAGGCAGTT | CACTCGACGATCACGCCCCT | 59.5 |
| *LOC_Os06g09240* | 300 | GGGCGTCGGTGATGGAGG | ATGCACAGATTATCTCGACGAACT | 57 |
| *LOC_Os06g18670* | 160 | AACCCCATCGCCAAGGACCTG | TCTTGAGGCGACGCACACCG | 58 |
| *LOC_Os06g39070* | 160 | GAGGAGATCCCGCCTACGGT | ACGATTCGAAGGAGGCCACG | 57 |
| *LOC_Os07g13810* | 160 | GCTGTATGCCAGCGCTACCC | ATGGGCACCCCCTCACAGA | 62 |
| *LOC_Os07g30690* | 160 | GTTGATACTCAACACCGCCGC | CCACGCCATGCAGCCATC | 57 |
| *LOC_Os07g30760* | 160 | GGATGGTGAGGGAGGCG | GCGTGTTGGATACGCGTG | 61.5 |
| *LOC_Os07g32620* | 160 | CGCTGATGGACGACGC | CGGATTTGAACGGCACC | 57 |
| *LOC_Os09g30980* | 160 | CACCCATGTCGGCTTCTCACG | GCACGCTCCTCCAACGACAC | 57 |
| *LOC_Os10g18510* | 160 | GACATCTGGCGGGTCGGG | CCTCCTGAACGCTCTCGTGT | 59 |
| *LOC_Os11g27370* | 160 | GTTTCAGCTCCGTCGTGGAGG | GCGTCATGGCCAAACCATCC | 57 |
